# COVIDanno, COVID-19 annotation in human

**DOI:** 10.3389/fmicb.2023.1129103

**Published:** 2023-07-11

**Authors:** Yuzhou Feng, Mengyuan Yang, Zhiwei Fan, Weiling Zhao, Pora Kim, Xiaobo Zhou

**Affiliations:** ^1^West China Biomedical Big Data Center, West China Hospital, Sichuan University, Chengdu, China; ^2^Med-X Center for Informatics, Sichuan University, Chengdu, China; ^3^School of Life Sciences, Zhengzhou University, Zhengzhou, China; ^4^Center for Computational Systems Medicine, School of Biomedical Informatics, The University of Texas Health Science Center at Houston, Houston, TX, United States; ^5^West China School of Public Health and West China Fourth Hospital, Sichuan University, Chengdu, China; ^6^McGovern Medical School, The University of Texas Health Science Center at Houston, Houston, TX, United States; ^7^School of Dentistry, The University of Texas Health Science Center at Houston, Houston, TX, United States

**Keywords:** COVID-19, *in vitro* models, infection state, expression, regulatory network

## Abstract

Severe acute respiratory syndrome coronavirus 2 (SARS-CoV-2), the etiologic agent of coronavirus disease 19 (COVID-19), has caused a global health crisis. Despite ongoing efforts to treat patients, there is no universal prevention or cure available. One of the feasible approaches will be identifying the key genes from SARS-CoV-2-infected cells. SARS-CoV-2-infected *in vitro* model, allows easy control of the experimental conditions, obtaining reproducible results, and monitoring of infection progression. Currently, accumulating RNA-seq data from SARS-CoV-2 *in vitro* models urgently needs systematic translation and interpretation. To fill this gap, we built COVIDanno, COVID-19 annotation in humans, available at http://biomedbdc.wchscu.cn/COVIDanno/. The aim of this resource is to provide a reference resource of intensive functional annotations of differentially expressed genes (DEGs) among different time points of COVID-19 infection in human *in vitro* models. To do this, we performed differential expression analysis for 136 individual datasets across 13 tissue types. In total, we identified 4,935 DEGs. We performed multiple bioinformatics/computational biology studies for these DEGs. Furthermore, we developed a novel tool to help users predict the status of SARS-CoV-2 infection for a given sample. COVIDanno will be a valuable resource for identifying SARS-CoV-2-related genes and understanding their potential functional roles in different time points and multiple tissue types.

## Introduction

SARS-CoV-2 poses a significant and widespread health threat. As of December 2022, there have been 642M confirmed cases of COVID-19, including 6.6M deaths, according to COVID-19 situation dashboard of World Health Organization (https://covid19.who.int). The host immune response plays a crucial role in the fight against viruses. However, host cell metabolisms can be altered by viral factors, immune regulatory factors, and various medicinal factors in the *in vivo* environment. Most of all, the human immune system is highly variable among individuals due to diverse factors, including different combinations of genetics/epigenetic factors (such as sex and age) and environmental factors. The human immune system is highly variable, making it difficult to grasp the key features as a whole. A good way to overcome these limitations is to infect target cells directly with SARS-CoV-2 *in vitro*. The *in vitro* models only include the viral factors, without the confounding variables present in the *in vivo* environment. It is also easy to control the experimental condition, obtain reproducible results, and monitor the progression of infection.

COVID-19 patients can present symptoms in multiple systems of the human body, including the respiratory, cardiovascular, gastrointestinal, hepatic, and ocular systems (Sridhar and Nicholls, 2021). Many studies have shown that SARS-CoV-2 can infect multiple tissues, such as the nose, lungs, eyes, stomach, intestines, heart, kidneys and liver (Lindner et al., 2020; Wichmann et al., 2020; Benvari et al., 2022; Brauninger et al., 2022; Chaurasia et al., 2022; Ramasamy, 2022). However, obtaining SARS-CoV-2-infected tissues from living COVID-19 patients, especially from specific tissues such as the heart, kidneys, intestines and liver, is difficult. Usually, the infected tissues are from autopsy cases. It is unclear what happened during the progression of the disease. RNA-seq data are collected from COVID-19 patients, who usually exhibit certain clinical symptoms that can be detected. However, these data lack information about the initial infection process (incubation period). *In vitro* models are useful for exploring the continuous infection progression and addressing immunologic drivers in the early stages of SARS-CoV-2 infection. A systematic comparison between *in vitro* models and *in vivo* conditions may provide novel and useful insights for improving COVID-19 therapeutics and drug development.

Currently, numerous *in vitro* models of multiple human tissues have been built to study COVID-19. To date, there are 11 COVID-19-related data resources and 4 databases that integrate publicly available COVID-19-related RNA-seq data (Satyam et al., 2021). However, the knowledge obtained from these databases is limited, and a comprehensive analysis is lacking. Most importantly, none of these databases focused on *in vitro* models infected with SARS-CoV-2. Although RNA-seq data from *in vitro* models of SARS-CoV-2 infection have accumulated, systematic translation/interpretation of these data is lacking. To address this gap, we integrated all existing RNA-seq datasets from SARS-CoV-2 *in vitro* models from Gene Expression Omnibus (GEO) (Barrett et al., 2013). In total, we collected 745 samples across 13 human tissues (brain, bronchi, eyes, heart, kidneys, large intestine, liver, lungs, nasal cavity, nerves, pancreas, small intestine, and stomach). We performed multiple bioinformatics analyses on the 4,935 significant DEGs, including gene group annotation, expression profiling, exon skipping event annotation, expression trajectory analysis, tissue-specific expression analysis, regulatory network analysis, drug and disease information integration, and curation of previous studies. We built a new database COVIDanno, COVID-19 annotation in human, available at http://biomedbdc.wchscu.cn/COVIDanno/. COVIDanno aims to provide resources and references for intensive functional annotations of the significant DEGs among different time points after COVID-19 infection from *in vitro* models. Additionally, COVIDanno provides a novel tool that enables users to predict infection status for a given SARS-CoV-2-infected sample through an unsupervised analysis method.

## Materials and methods

### Data quality control and reads alignment

The raw RNA-seq data (fastq files) of SARS-CoV-2 *in vitro* models were downloaded from GEO. Fastp (Chen et al., 2018) was used to perform quality checks of fastq files. The quality checked reads were then mapped to the Ensembl human reference genome (GRCh38 release 103; Yates et al., 2020) using STAR aligner (Dobin et al., 2013) and SARS-CoV-2 reference genome (GenBank: NC_045512.2) using Bowtie2 (Langmead and Salzberg, 2012). After quality control and alignments, read counts were summarized using the featureCounts function of the Subread package (Liao et al., 2014).

### Sample relationship analysis

The raw read counts of the RNA-seq data were normalized using the variance stabilizing transformation (VST) after mapping to the human reference genome and SARS-CoV-2 reference genome. The VST normalized counts were then used to generate sample correlation results using the Pearson correlation coefficient and perform principal component analysis (PCA).

### Differential gene expression analysis

To perform differential gene expression analysis, we first removed the SARS-CoV-2 viral transcripts. DEseq2 (Love et al., 2014) was then used to identify the DEGs between SARS-CoV-2-infected and mock-treated samples. Next, we performed various bioinformatics/computational biology studies for these DEGs.

### Detection of alternative splicing events

rMATS (Shen et al., 2014) was used to identify the differential alternating splicing (DAS) events between SARS-CoV-2-infected and mock-treated samples and obtain percent spliced-in (PSI) values of individual samples. Five types of DAS events were identified, including exon skipping (ES), alternative 5′ splice site (A5SS), alternative 3′ splice sites (A3SS), mutually exclusive exon (MXE), and intron retention (RI). PSI values of SARS-CoV-2-infected and mock-treated samples were corrected for batch effect using the removeBatchEffect function in limma (Ritchie et al., 2015).

### Functional enrichment analysis for DEGs and differential exon skipping events

We performed enrichment analysis using Kyoto Encyclopedia of Genes and Genomes (KEGG) (Kanehisa and Goto, 2000) and Gene Ontology (GO) (Blake et al., 2015) pathways for DEGs (*p*.adj < 0.05 and |log2FC| > 1) and differential exon skipping events (FDR < 0.05 and |∆ PSI| > 0.1) by the Enrichr tool (Kuleshov et al., 2016).

### Landscaping of gene expression and PSI values

To gain insight into the gene expression patterns, counts were then normalized using the TMM method in edgeR (Robinson et al., 2010). These TMM-normalized counts were then transformed into TMM normalized log-CPM values. Finally, the batch-corrected TMM normalized log-CPM gene expression values of SARS-CoV-2-infected and mock-treated samples were used to visualize the landscape of individual genes across 136 datasets *via* heatmaps. For PSI patterns, batch-corrected PSI values of SARS-CoV-2-infected and mock-treated samples were used to visualize the landscape of individual exon skipping events across 136 datasets *via* heatmaps. We corrected the batch effect using the removeBatchEffect function in the limma package (Ritchie et al., 2015).

### Construction of genetic regulatory networks of transcription factors for COVID-19 infection DEGs

We used PANDA (Glass et al., 2013), the baseline method in netzoo, to construct gene regulatory networks between transcription factors (TFs) and their target genes by combining information from gene expression, protein–protein interaction, and transcription factor regulatory data. First, we downloaded position weight matrices (PWMs) for *Homo sapiens* motifs from CIS-BP (version 2.0) (Weirauch et al., 2014). Then, we mapped the PWMs to promoter regions using FIMO (Grant et al., 2011). The sequence motifs of 940 TFs were mapped into the promoter region ranging from –750 to +250 around the transcription start site (TSS) with a significant value of *p* less than 10E-5 (Supplementary Figure S1A). Finally, we used PANDA (Glass et al., 2013) to estimate population-based networks by integrating 940 TFs, gene expression profiles, and protein–protein interactions (StringDB) (Szklarczyk et al., 2015). To compare these regulatory networks between SARS-CoV-2-infected and mock-treated samples, we used panda.diff.edges function with a default threshold value 0.8 for differential TF-gene edges.

### Construction of alternative splicing regulatory networks in response to SARS-CoV-2 infection

We used PANDA (Glass et al., 2013) to infer alternative splicing (AS) regulatory networks between RNA-binding proteins (RBPs) and their target exon skipping events. First, we downloaded PWMs for *Homo sapiens* motifs from CisBP-RNA (version 0.6) (Weirauch et al., 2014) and mapped the PWMs to the skipped exon regions using FIMO (Grant et al., 2011). The sequence motifs of 73 RBPs mapped with a value of *p* less than 10E-4 within 4 skipped exon regions (referring rMAPS2; Hwang et al., 2020) (Supplementary Figure S1B). Then, we used PANDA (Glass et al., 2013) to estimate population-based networks by integrating 73 RBPs, PSI values of exon skipping events, and protein–protein interactions (StringDB) (Szklarczyk et al., 2015). To compare these network models between SARS-CoV-2-infected and mock-treated samples, we used panda.diff.edges function with a default threshold value 0.8 for differential RBP-ES edges.

### Gene group annotation (immune relatedness, sex relatedness, aging relatedness, and tissue specificity)

For further dissecting the DEGs, we overlapped our DEGs with specific gene groups such as immune-related genes, sex-related genes, age-related genes, and tissue-specific genes. Immune-related genes were extracted from InnateDB (Breuer et al., 2013) and immune response-related pathways from KEGG (Kanehisa and Goto, 2000) and GO (Blake et al., 2015). Sex-related genes were extracted from SAGD (Shi et al., 2019). Aging-related genes were extracted from GenAge (de Magalhaes and Toussaint, 2004) and Aging Atlas (Liu G. H. et al., 2021). Tissue-specific genes were extracted from TissGDB (Kim et al., 2018).

### Drug and disease information

Drug-target interactions (DTIs) were extracted from DrugBank (Wishart et al., 2018) (May 2022, version 5.1.9). All drugs were grouped using Anatomical Therapeutic Chemical (ATC) classification system codes. Disease-related genetic information was extracted from a database of gene-disease associations (DisGeNet, May 2022, version 7.0) (Pinero et al., 2017).

### Curation of PubMed articles

To understand the current research progress, we used RISmed (version 2.3.0) to retrieve the related literature related to the DEGs. PubMed’s literature query was performed in August 2022 using the keywords for DEG (gene symbol, synonyms of gene symbol). Taking ACE2 as an example, the searching keywords used were ‘(COVID-19 [Title/Abstract] OR SARS-CoV-2 [Title/Abstract]) AND (ACE2 [Title/Abstract] OR ACEH [Title/Abstract])’.

### Infection status prediction of SARS-CoV-2-infected samples (inferred time)

Viral infection can trigger host pattern recognition receptors (PRRs) to initiate antiviral innate immune responses. The intracellular signaling cascades triggered by these PRRs lead to altered expression of cytokines and chemokines against the virus. Here, we defined the immune response genes, which are enriched in PRRs, cytokines, and chemokines-related pathways, to explore the infection severity of SARS-CoV-2 in the infected samples. We identified 891 immune response genes by integrating data from 132 paired datasets (742 samples). To assess the richness of immune response genes, rarefaction curves were generated by randomly re-sampling the pool of N datasets several times and then plotting the average number of immune response genes identified in each dataset.

To minimize the impact of batch effects and tissue difference, we performed gene expression analysis of 132 paired datasets under 4 matched conditions, including GEO accession number, sub-tissue type, hours post-infection (hpi) value, multiplicity of infection (moi) value (Supplementary Figure S2). We used log2FC values of 891 immune response genes from 132 paired datasets to explore the severity of SARS-CoV-2 infection. Monocle2 (Qiu et al., 2017), which can measure cell transition from one state to another in disease using gene expression data, was used for pseudotime inference. We studied transcriptional heterogeneity in immune responses by clustering 132 paired datasets based on their individual position on the pseudotime, following a previous study (Meistermann et al., 2021). To do this, a k-means (*k* = 8) was performed to separate the 132 paired datasets into 8 clusters, with each cluster containing at least 3 datasets and datasets with sub-branch belonging to the same cluster. We performed projective clustering (k-means) based on the position of datasets on the pseudotime. Clusters are ordered according to their mean pseudotime. The information of 132 datasets used in the tool is shown in Supplementary Figure S3 and Supplementary Table S1.

### A tool for exploring the infection status of a given SARS-CoV-2-infected sample

For pseudotime prediction of infected conditions, we divided 132 paired datasets (742 samples) into 8 continuous infection states (Supplementary Figure S3). When a given SARS-CoV-2-infected sample was input, we combined it with the 132 paired datasets and followed the same procedure to predict the infection pseudotime. Subsequently, we compare the pseudotime position distance for a given SARS-CoV-2-infected sample with the 8 positions representing the infection state clusters. The infection severity of the given SARS-CoV-2-infected sample was determined based on its similarity to the closest infection state cluster.

### Expression trajectory analysis of 14 sub-tissues to infer behaviors of individual DEGs over time

TMM normalized log-CPM data of SARS-CoV-2-infected samples were used to explore expression trajectory patterns at different hours post-infection (real infection time in experiments) and infection state clusters (inferred time). Normalized data were corrected for batch effects using the removeBatchEffect function in limma (Ritchie et al., 2015). Sub-tissues, including at least 2 time points post-infection and with the same moi values, were used to perform expression trajectory analysis.

### Tissue-specific expressed genes across SARS-CoV-2 infection state (inferred time)

To identify tissue-specific expressed genes in SARS-CoV-2-infected samples with the same infection state, we generated a gene list by evaluating z-scores based on the expression levels of the genes. Here, a *z*-score equal to N represents more than N standard deviations greater than the mean expression in all tissues. For the appropriate number of genes, we set a threshold of 1.3 for the *z*-score in the expression data for each infection state.

### Exploring disease progression of different tissue types

We inspected a scatter plot of the infection state compared with the hpi value in the same SARS-CoV-2-infected samples. These samples had the same GEO accession number, sub-tissue type, moi value, and multiple hpi values. The scatter plot showed that the relationship between infection states and hpi values apparently follows a linear regression model with logarithmic transformations. The model can be represented as follows:


(1) $$Y=\beta 0+\beta 1^{*}\log\;\left(X+1\right).$$

where Y is the infection state of a paired dataset, which is the dependent variable. X is the hpi value of a paired dataset. The goodness of fit is quantified by *R*^2^, which is the square of the correlation *r* between percentage infection states and hpi values.

## Results

### Database overview

**Figure 1:**
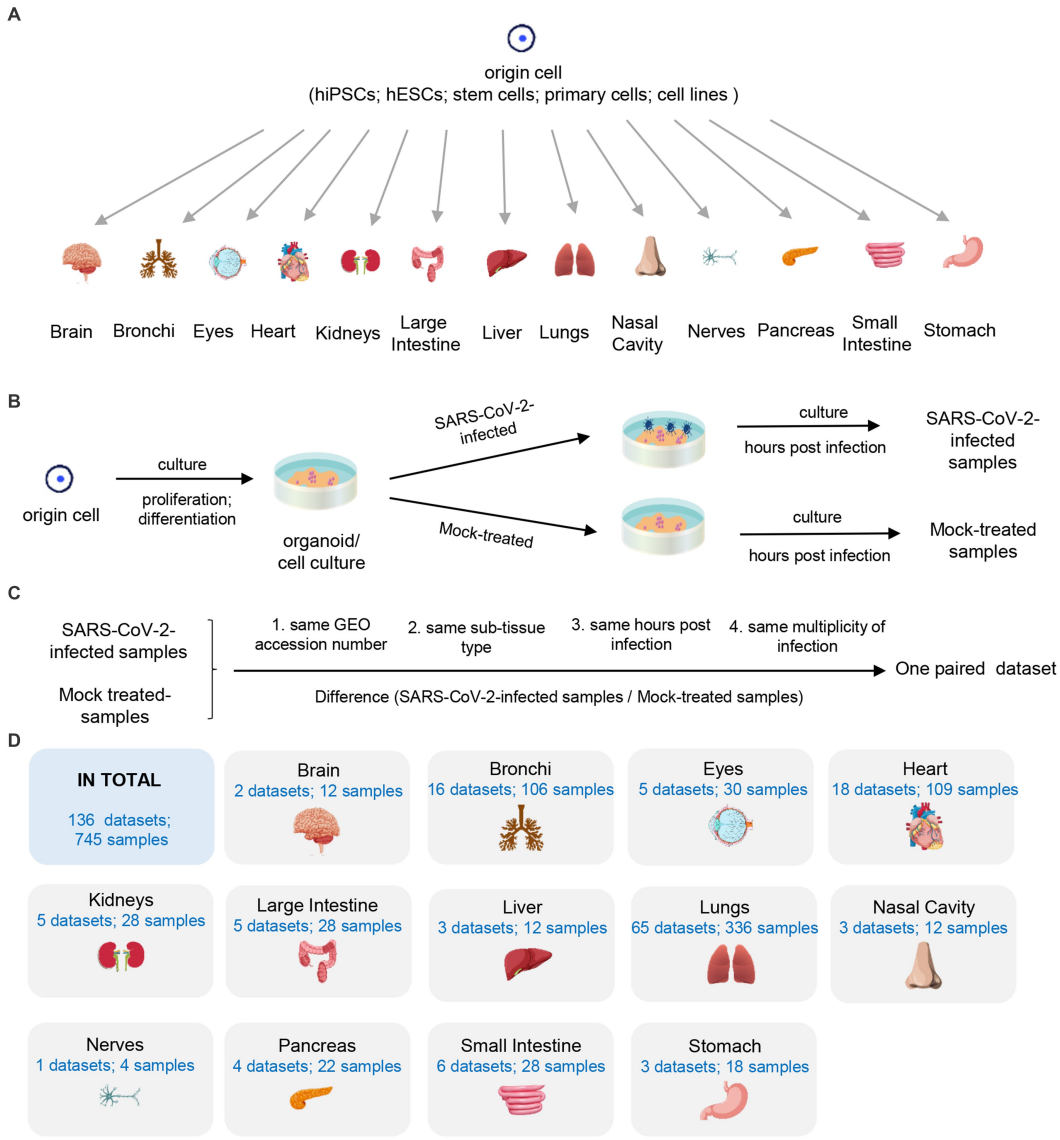
Description of used 136 datasets. **(A)** The origin of 13 human tissues. **(B)** The generating of SARS-CoV-2-infected and mock-treated samples. **(C)** The definition of the paired dataset. **(D)** Summary of collected samples.

We manually collected all available RNA-seq datasets of SARS-CoV-2 *in vitro* models from GEO database. First, we curated samples into paired datasets by matching each SARS-CoV-2-infected sample with its corresponding mock-treated samples. The criteria for pairing included the same GEO accession number, sub-tissue type, hpi value and moi value (Figures 1B,C). Next, we filtered the paired datasets with the following two criteria: (i) the dataset should contain both SARS-CoV-2-infected samples and their corresponding mock-treated samples with same conditions (GEO accession number, sub-tissue type, hpi value, and moi value); (ii) each group (SARS-CoV-2-infected or mock-treated group) should consist of at least two independent biological replicates to minimize variability. Finally, we collected a total of 136 paired datasets consisting of 745 samples from 13 human tissues, including brain, bronchi, eyes, heart, kidneys, large intestine, liver, lungs, nasal cavity, nerves, pancreas, small intestine, and stomach (Figures 1A,D). A comprehensive list of all datasets used in this study is shown in Supplementary Table S1.

**Figure 2:**
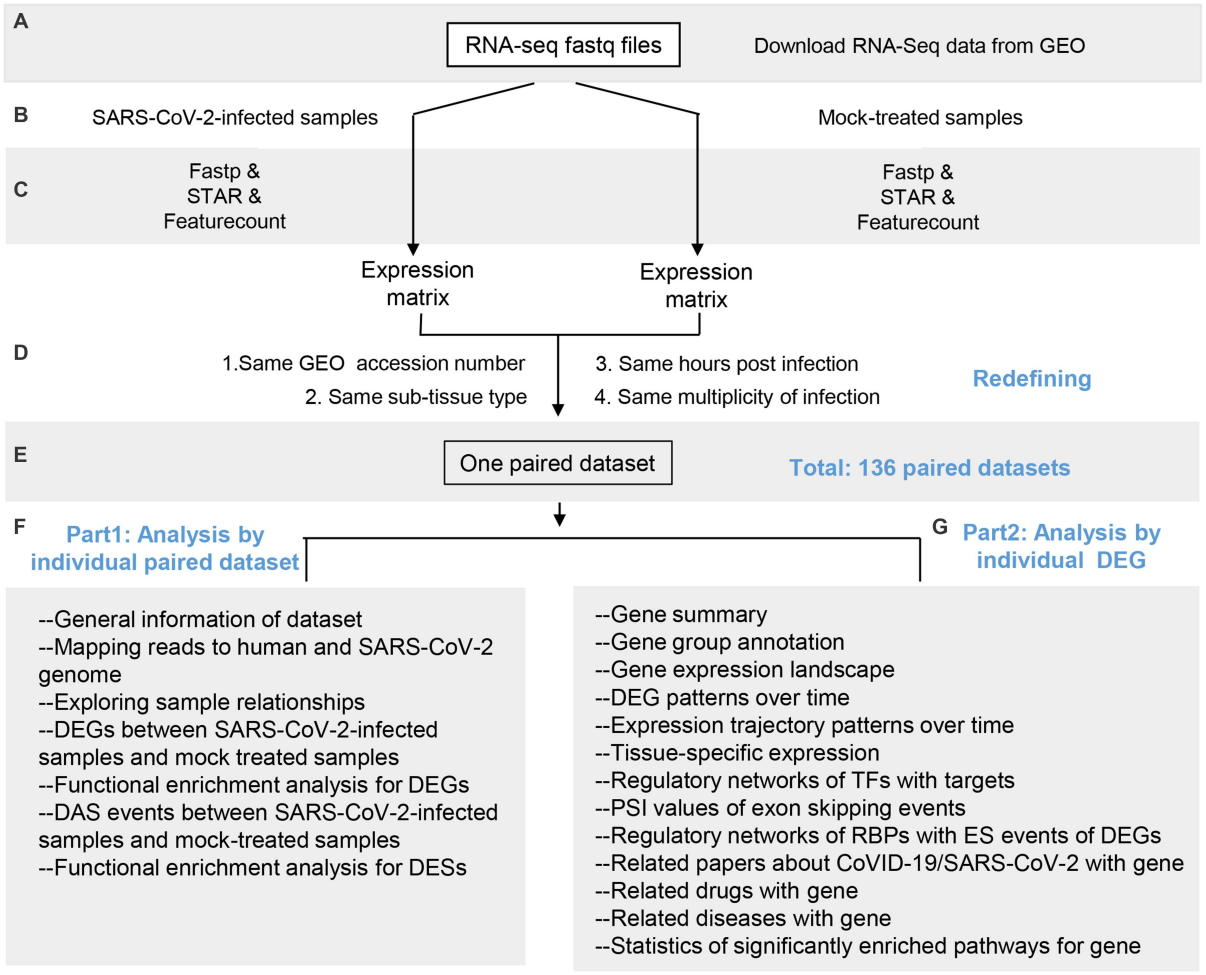
Overall schema of COVIDanno annotation pipeline. **(A–C)** Data collection, quality control, and alignment. **(D,E)** Redefining 745 samples into 136 paired datasets by matching SARS-CoV-2-infected samples with corresponding mock-treated samples based on 4 conditions. **(F)** Main categories for 136 individual datasets. **(G)** Main categories for 4,935 individuals significant DEGs by integrating 136 individual datasets.

The overall schema of COVIDanno is represented in Figure 2. The COVIDanno consists of 3 parts. Firstly, we performed differentially expressed analysis on these 136 paired datasets. Four thousand nine-hundred and thirty five were identified (*p*.adj < 0.001 and |log2FC| > 2). Subsequently, we performed diverse bioinformatics/computational biology studies on these DEGs. The main features of COVIDanno are summarized below, and other features can be found through our website link.

For each of the 136 individual datasets, we performed differentially expressed genes analysis (COVID-19 infection DEGs) and differential alternative splicing analysis (COVID-19 infection DESs) between SARS-CoV-2-infected and mock-treated samples. We then performed functional enrichment analyses on these DEGs and DESs to provide insight into the cellular working context after the COVID-19 infection. Overall, we identified a total of 4,935 DEGs associated with at least 3 GEO resources.

For 4,935 significant DEGs, individual genes were integrated with relevant gene groups (i.e., immune relatedness, sex relatedness, aging relatedness, and tissue specificity). We provided the expression landscape and exon skipping events values across 136 datasets. Expression trajectory analysis of 14 sub-tissues provided the inferred behaviors of individual DEGs over time. Tissue-specific expression analysis revealed tissue-specific changes during SARS-CoV-2 infection. The TF-gene and RBP-ES regulatory networks identified potential regulators for COVID-19 infection DEGs. In related drug analysis, we found that 903 COVID-19 DEGs were targeted by 3,577 FDA-approved drugs. Additionally, through related diseases analysis, we identified 3,801 COVID-19 DEGs reported in 19,189 diseases. We performed a curation of 4,935 genes regarding their expression in the COVID-19 infection samples by PubMed search. Among them, 1,704 genes have been reported to associate with COVID-19 progression.

Furthermore, through our study, we developed a novel online tool to predict the infection status for a given sample through an unsupervised analysis method. This approach was validated by applying it to multiple datasets from previous studies.

### Analysis of differential gene expression and their regulatory networks at different time points of COVID-19 infection in *in vitro* models

From the DEG analysis, we observed significant changes in host gene expression landscape following SARS-CoV-2 infection. Further analysis of these changes will be helpful in developing new avenues for antiviral therapies. In the 136 individual datasets, we performed differential gene expression analysis between SARS-CoV-2-infected and mock-treated samples. In order to reduce background noise and generate reliable a set of DEGs, we implemented a series of stringent filters (*p*.adj < 0.001, |log2FC| > 2, DEGs identified at least 3 GEO resources). After screening, we identified 4,935 genes with significant expression changes by integrating DEGs from 136 individual datasets. The distributions of 4,935 DEGs are shown in Supplementary Figure S4. More than 4,200 genes show a significant difference in at least 2 tissue types. Lungs and heart have the most significant number of differentially expressed genes, which is in line with findings that SARS-CoV-2 mainly affects the lungs and heart in COVID-19 patients (Bavishi et al., 2020; Huang et al., 2020). A gene summary of the 4,935 DEGs is shown in Supplementary Table S2. We then performed GO and KEGG pathway enrichment analysis for 136 individual datasets to investigate the functions related to biological responses or processes during SARS-CoV-2 infection. Overall, we found that the up-regulated DEGs were mainly enriched in the biological processes related to ‘transcription regulation’, ‘cytokine’ and ‘anti-virus immune response’ -related pathways. In previous studies, various cytokines and chemokines have been observed in different stages of COVID-19 and act as independent risk factors for disease severity and mortality. However, the molecular pathogenesis underlying COVID-19-associated cytokine storm is unknown. These DEGs, identified through *in vitro* models provide a unique advantage in understanding the immune activation process and the severe-to-critical symptom (cytokine storm) in COVID-19 patients (Wang J. et al., 2020).

The importance of transcriptional regulation of host genes in innate immunity against viral infection has been widely recognized. Construction of TF regulatory networks can help identify potential upstream TFs for therapeutic targeting. For 14 sub-tissues, which have at least 3 individual datasets, we constructed TF regulatory networks for both SARS-CoV-2-infected and mock-treated samples. In addition, we performed differential network analysis between SARS-CoV-2-infected and mock-treated samples.

### Alternative splicing events among different time points of the COVID-19 infection in human *in vitro* models and their regulatory networks

AS is a crucial post-transcriptional mechanism enabling single genes to produce structurally and functionally distinct protein isoforms (Wang et al., 2008). Host splicing changes have been observed during infection with RNA viruses such as reovirus (Boudreault et al., 2016), Herpes simplex virus −1 (HSV1) (Ku et al., 2011), dengue virus (Sessions et al., 2013), zika virus (Hu et al., 2017) and SARS-CoV-2 (Arora et al., 2020; Banerjee et al., 2020). However, a systematic and intensive analysis of AS in COVID-19 is still lacking. For 136 individual datasets, we did DAS analysis between SARS-CoV-2-infected and mock-treated samples. Exon skipping events are the most prevalent type of alternative splicing events in the human genome, and are well represented in the databases. We performed GO and KEGG pathway enrichment analyses to gain insights into the biological pathways associated with the genes undergoing exon skipping. Our analysis revealed that these genes, which exhibit exon skipping events, were enriched in ‘transcription regulation,’ ‘protein modification’ and ‘mRNA processing’-related biological pathways. From our analysis, we identified 1,443 exon skipping events of 767 DEGs, each of which was identified from at least 3 GEO resources. Notably, our findings revealed the involvement of specific genes in important biological processes. For instance, IFI16 plays a role in the negative regulation of viral genome replication and can initiate different innate immune responses (Karlebach et al., 2022). Additionally, alternative splicing of MX1 supports rather than restricts viral infection (Ku et al., 2011; De Maio et al., 2016). Our findings provide further insights into the complex molecular mechanisms associated with viral infections and host responses, expanding our understanding of alternative splicing events in COVID-19.

Recently, post-transcriptional regulatory mechanisms have gained appreciation as an additional and important layer of regulation to fine-tune host immune responses. RBPs are a group of proteins that bind to mRNAs or non-coding RNAs, playing diverse roles in post-transcriptional processing and RNA regulation (Li et al., 2014). Therefore, we construct RBP regulatory networks to investigate the changes and regulation of alternative splicing events. For 14 sub-tissues with at least 3 individual datasets per tissue, we constructed RBP regulatory networks for both SARS-CoV-2-infected and mock-treated samples. We then performed differential network analysis between SARS-CoV-2-infected and mock-treated samples in order to identify potential regulatory changes associated with SARS-CoV-2 infection.

### Important gene group annotations (i.e., immune, sex, aging, and tissue specificity)

Clinical experience to date has shown that COVID-19 is highly heterogeneous, ranging from asymptomatic, mild, moderate, to severe and critical. Host factors, including age and sex, are key determinants of disease severity and progression (Alwani et al., 2021; Chen et al., 2021; Hobbs et al., 2021). The exaggerated immune response induced by the cytokine storm is an independent risk factor for disease severity and mortality. Furthermore, multiple tissue types could be susceptible to SARS-CoV-2 and COVID-19 patients presenting symptoms in multiple systems (Yang et al., 2013; Hong et al., 2020; Jin et al., 2020; Qi et al., 2020). To gain insights into the molecular basis of COVID-19, we analyzed the overlap between 4,935 significant DEGs and specific gene groups, including immune-related genes, sex-related genes, age-related genes, and tissue-specific genes. Our analysis identified 560 immune-related genes, 230 sex-associated genes, 170 aging-related genes, and 718 tissue-specific genes within the set of significant DEGs (Supplementary Figure S5). Among them, 6 genes were present in all four gene groups. All of the 6 intersected genes have been reported to associate with COVID-19, including FGFR3 (Hachim et al., 2021), TP63 (Delorey et al., 2021), CXCL2 (Livanos et al., 2021), CCL20 (Chua et al., 2020), IL1B (Chua et al., 2020) and CXCL8 (Zheng et al., 2021). Annotation of these gene groups provides valuable insights into their functional relevance in the context of COVID-19.

### Infection status prediction of SARS-CoV-2-infected samples (inferred time)

Currently, there are accumulated RNA-seq data generated from SARS-CoV-2-infected *in vitro* models. However, there is a lack of systematic evaluation of the infection severity of these samples. It is difficult to compare SARS-CoV-2-infected samples from different studies with different tissue types, hpi values and moi values. Additionally, systematic evaluation of infection severity in SARS-CoV-2-infected samples is lacking. For example, although GSE151513 contains 6 infection time points (0–12 h), there is no obvious difference between the degree of infection (Supplementary Figure S6C). To better understand the continuous infection progress and severity of SARS-CoV-2-infected samples, we did infection state prediction by pseudotime analysis.

**Figure 3:**
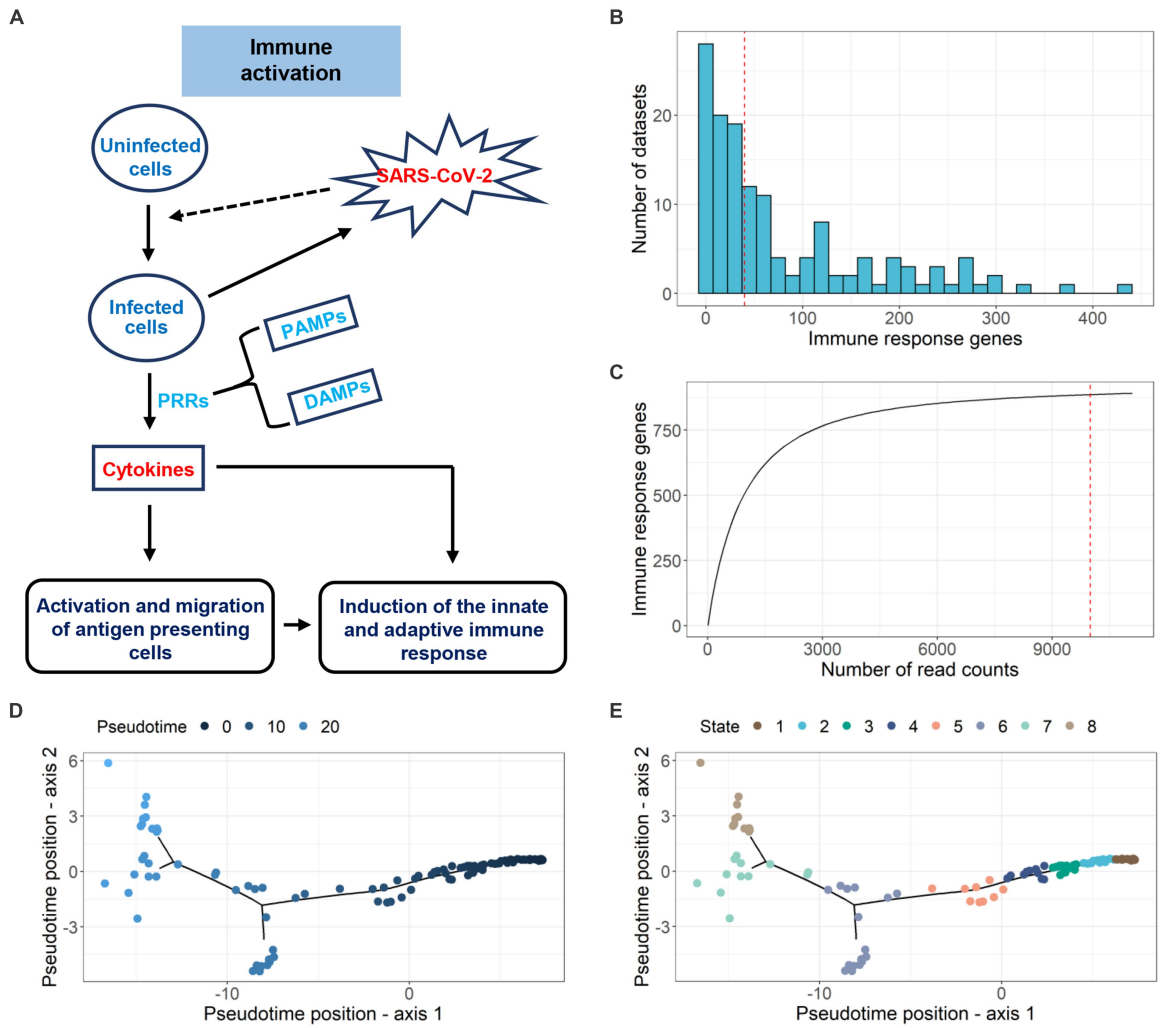
Pseudotime inference of SARS-CoV-2-infected samples. **(A)** Schematic diagram of the immune activation process. **(B)** The distribution of differential immune response genes (adj.*p* < 0.05 and |log2FC| > 1) in 132 datasets. **(C)** Investigating the immune response genes richness using rarefaction curve. **(D)** Pseudotime inference for 132 paired datasets using DEGs. **(E)** Infection state prediction for 132 datasets.

Viral infection triggers host PRRs to initiate antiviral innate immune responses by pathogen-associated molecular patterns (PAMPs) or danger-associated molecular patterns (DAMPs) (Carty et al., 2021; Li and Chang, 2021; Zheng, 2021). The intracellular signaling cascades triggered by these PRRs lead to the activation of diverse transcriptional factors that regulate the expression of cytokines and chemokines. Such cytokines and chemokines play important roles in host protection, activation and migration of antigen-presenting cells, and induction of adaptive immune responses. The schematic diagram of the immune activation process is shown in Figure 3A. In our study, we extracted 891 immune response genes from the immune activation process by integrating 132 paired datasets (742 samples). The distribution of immune response genes in the datasets is illustrated in Figure 3B. The rarefaction curves represent the immune response gene richness for a given number of individual datasets. A plateau in the rarefaction curves indicates a good representation of immune response genes (Figure 3C). Even with the increase in the number of datasets, the number of immune response genes did not change much. Subsequently, we divided 132 paired datasets into 8 continuous infection states according to gene expression changes of 891 immune response genes during SARS-CoV-2 infection using an unsupervised analysis method (Figures 3D,E). We validated this approach by applying it to datasets with multiple hpi values but the same GEO accession number, sub-tissue type and moi value. Seventy-four datasets with multiple hpi values showed that as the hpi value (real infection time in experiments) increased, the infection state (inferred time) increased or remained the same (Supplementary Figure S6). The information of 132 datasets used in the tool can be found in Supplementary Figure S3 and Supplementary Table S1.

### A tool for exploring the infection status of a given SARS-CoV-2-infected sample

We developed a novel online tool using 132 datasets (724 samples) to explore the severity of SARS-CoV-2-infected samples *in vitro*. When a given SARS-CoV-2-infected sample was input, we combined it with the 132 paired datasets and followed the same procedure to predict the infection pseudotime. Subsequently, we compared the pseudotime position distance of the given SARS-CoV-2-infected sample with a center position of 8 infection state clusters. The given SARS-CoV-2-infected sample was assigned to the closest infection state cluster. Seventy-four datasets with multiple hpi (hours post-infection) values were analyzed. Our results revealed a consistent relationship between hpi and inferred infection state (Supplementary Figure S6). To further validate the performance of the tool, we applied it to the datasets (BI_10 and BI_11 from GSE196464) that were not used during tool development. As shown in Supplementary Figure S7, as the hpi value increased (24–72 hpi), the infection state also increased (state 5 to state 6). These results were stable and exhibited consistent patterns. The detailed information of 132 datasets used in the tool is shown in Supplementary Figure S3 and Supplementary Table S1.

### Application of COVIDanno to enhance understanding of COVID-19 anosmia symptom

**Figure 4:**
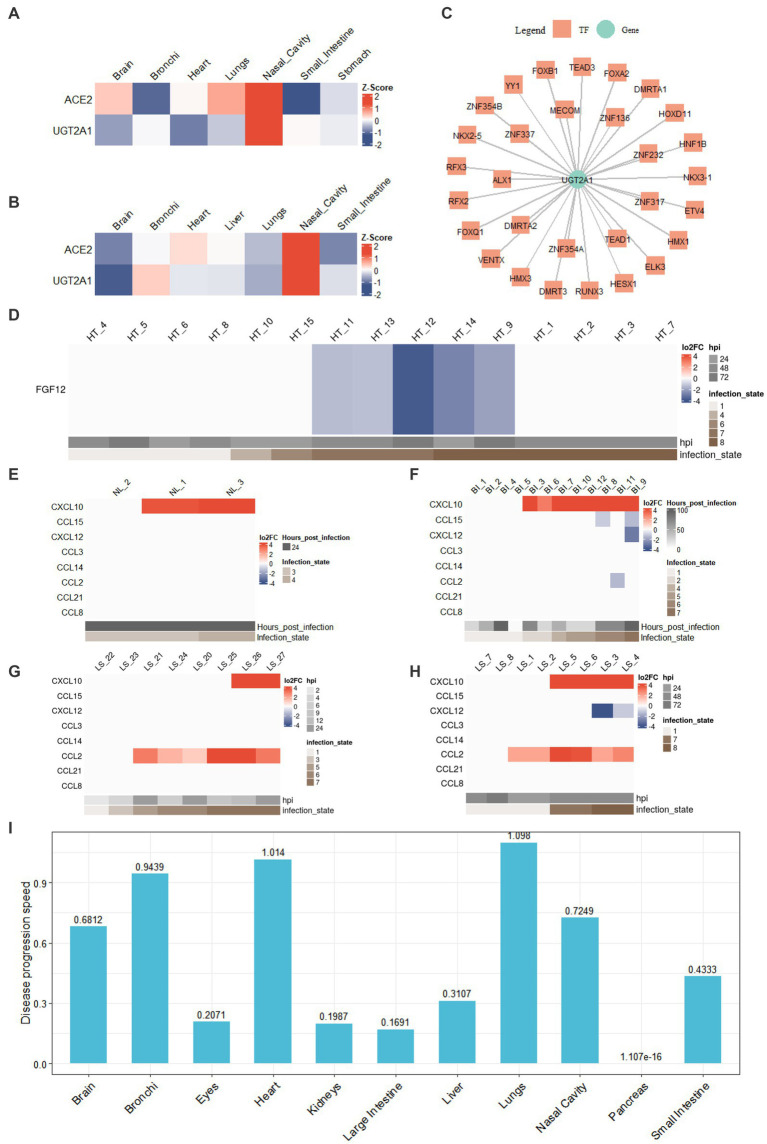
COVIDanno analyses. **(A)** Tissue-specific genes in the infection state 3 across tissues. **(B)** Tissue-specific genes in the infection state 4 across tissues. **(C)** TF-gene regulatory network composed of UGT2A1 gene and associated top 30 TFs. **(D)** DEG heatmap across infection time as an example of FGF12 (adj.*p* < 0.05 and |log2FC| > 1). DE chemokines (adj.*p* < 0.05 and |log2FC| > 1) in **(E)** nasal cavity tissue. **(F)** Bronchi tissue. **(G)** A549 cell line of lungs. **(H)** Lung organoid of lungs. **(I)** Disease progression across 11 tissue types using the regression model.

Anosmia (loss of smell) is a common symptom of COVID-19. Recent studies have shown that non-neuronal supporting cells of the human olfactory epithelium express ACE2, which is necessary for SARS-CoV-2 infection. In our studies, we observed high expression of ACE2 in nasal cavity samples (Supplementary Figure S8). For SARS-CoV-2-infected nasal cavity samples, we identified 212 tissue-specific expressed genes in all infection states (state 3, state 4) with z-score greater than the threshold 1.3. For instance, ACE2 and UGT2A are among the 212 genes, and their expression patterns are shown in Figures 4A,B. Four of 212 tissue-specific expressed genes have been reported to associate with smell in previous studies, including UGT2A1 (Leclerc et al., 2002; Neiers et al., 2021), ACE2 (Gupta et al., 2021), KISS1 (Valdes-Socin et al., 2014), and GRM2 (Kim et al., 2020). In particular, UGT2A1 has been reported to associate with COVID-19-related loss of smell and taste in multiple studies (Khan et al., 2021; Hendaus, 2022; Shelton et al., 2022). In our studies, UGT2A1 was significantly down-regulated (log2FC < −2 and adj.*p* < 0.001) in the SARS-CoV-2-infected nasal cavity samples. Through the genetic regulatory network analysis, we identified the transcription factors associated with UGT2A1 (Figure 4C). HESX1, with a high probability of regulating UGT2A1, has been previously reported to be associated with smell (Valdes-Socin et al., 2014). Transcription factors TEAD1 and FOXA2 are associated with taste and were found to regulate UGT2A1 (Inamdar et al., 1993; Golden et al., 2021). The loss of smell and taste is well-known and often the sole COVID-19 symptom. COVIDanno provides valuable insights by analyzing genetic regulatory networks and identifying potential regulatory genes associated with specific symptoms. By deciphering the intricate interplay between genes, transcription factors, and regulatory pathways, COVIDanno aids in uncovering the molecular basis of symptoms like anosmia.

### Application of COVIDanno to enhance understanding of arrhythmia symptom in COVID-19

Growing evidence shows that arrhythmias are also one of the major complications of COVID-19. A previous report from Wuhan, China, revealed that 16.7% of hospitalized and 44.4% of ICU COVID-19 patients experienced cardiac arrhythmias (Wang D. et al., 2020). In a cohort study conducted in New York, atrial arrhythmias rates were 17.7% in mechanically ventilated COVID-19 patients and 1.9% in non-invasive ventilation COVID-19 patients (Goyal et al., 2020). SARS-CoV-2 virus load was detected in the myocardial tissue and showed signs of viral replication within the myocardial tissues in autopsy cases (Lindner et al., 2020; Brauninger et al., 2022). This is in line with the finding that ACE2 is expressed within myocardial cells (Nicin et al., 2020), and myocardium is infected by SARS-CoV (Oudit et al., 2009).

Fibroblast growth factor (FGF) homologous factors (FHFs), a subfamily of FGF proteins (FGF11–FGF14), are expressed predominantly in excitable cells (Goldfarb, 2005) and can modulate both Na+ and Ca2+ channels (Wang et al., 2011; Hennessey et al., 2013b). Among them, FGF12 has been reported to associate with arrhythmias (Hennessey et al., 2013a; Li et al., 2017). In our studies, we investigated the expression of FGF12 in the context of SARS-CoV-2 infection. We observed that FGF12 was significantly down-regulated in late infection state 7 of the heart and gradually recovered in infection state 8 (Figure 4D). The continuous infection state was validated by multiple GEO resources (Supplementary Figure S6). The datasets from 4 individual studies (GSE162736, GSE150392, GSE184715, and GSE151879) showed a significant down-regulation of FGF12. However, in autopsies of COVID-19 patients, no significant changes in FGF12 expression were observed in cardiomyocytes (Lindner et al., 2020; Brauninger et al., 2022). Our result also showed a recovery in FGF12 expression in infection state 8, consistent with the reports (Figure 4D). Obtaining SARS-CoV-2-infected tissues from living COVID-19 patients, particularly in specific tissues such as the heart, kidneys, intestines and liver, is difficult. COVIDanno can help explore the continuous progression of SARS-CoV-2 infection.

### Application of COVIDanno to explore the biomarkers associated with disease severity of COVID-19 in the respiratory tract

Prior studies have demonstrated that immunologic dysfunction is a key factor underlying severe illness in COVID-19 patients. Elevated levels of multiple cytokines/chemokines have been observed in acutely severe/critically ill patients with COVID-19. Specifically, CCL2 and CXCL10 have been associated with an increased risk of death and poor prognosis in COVID-19 patients (Chen et al., 2020; Uranga-Murillo et al., 2022). Chua et al. found that CCL2 and CXCL10 were predominantly expressed in monocyte-derived macrophages (moMa) and non-resident macrophages (nrMa) within the respiratory tract (Supplementary Figure S9A; Chua et al., 2020). Macrophages have been found to play a crucial role during SARS-CoV-2 infections (Grant et al., 2021; Sefik et al., 2022). The expression of chemokine receptors (CCR1, CCR5, CXCR4) on moMa and nrMa was significantly altered in COVID-19 patients (Supplementary Figure S9B; Chua et al., 2020). Chemokines secreted in the initial phase recruit inflammatory innate and adaptive immune cells, resulting in an exaggerated inflammatory immune response. To explore the immunologic drivers within the respiratory tract, we analyzed the expression profiles of 7 chemokines (CCL2, CCL3, CCL8, CCL14, CCL15, CCL21, CxCL12) and their corresponding receptors (CCR1, CCR5, CXCR4). Detailed information of ligand-receptor pairs can be found in Supplementary Figure S9C.

As shown in Figures 4G,H, we observed a significant up-regulation of the chemokine CCL2 in the late infection states of lungs in 6 individual studies (GSE155241, GSE148697, GSE160435, GSE157057, GSE147507, and GSE184536). Increased expression of CCL2 during the initial phase of COVID-19 was also reported previously (Blanco-Melo et al., 2020). However, no increased expression of these 7 chemokines was observed in nasal cavities or bronchi tissues (Figures 4E,F), suggesting that SARS-CoV-2-infected cells in the upper respiratory did not secrete many chemokines to recruit moMa or nrMa. In contrast, SARS-CoV-2-infected lung cells secreted a high-level of CCL2 to recruit moMa. This is in line with the findings that early and effective immune responses in the upper respiratory tract limit (Ramasamy, 2022). Furthermore, we identified potential TFs with regulatory roles in the expression of CCL2 and CXCL10, such as STAT1, STAT3, IRF1, etc. (Supplementary Figures S9D,E). Khokhar et al. also reported that TFs STAT1 and STAT3 are potential regulators of CCL2, while TFs IRF1, IRF3, IRF7, and RELA are potential regulators of CXCL10 in a COVID-19 study (Khokhar et al., 2022). These findings provide important insights into the regulatory mechanisms of chemokine expression during SARS-CoV-2 infection, which may have implications for developing therapeutic strategies targeting specific regulatory genes. Therefore, COVIDanno can be a useful resource for addressing immunologic drivers and exploring potential regulatory factors in the early stages of SARS-CoV-2 infection.

### Application of COVIDanno to explore the disease progression of different tissue types

We applied linear regression models with logarithmic transformations to multiple datasets with continuous infection time from the same study. The *R*^2^ and *p-*values suggest a goodness of fit by using this model (Supplementary Figure S10). The slope coefficient β1 represents the rate of disease progression. However, usually, there are multiple moi values and sub-tissue types within one tissue, and both factors can influence the disease progression. Therefore, we fit the linear model for each tissue with different moi values and sub-tissue types to provide an overview of 11 tissues (brain, bronchi, eyes, heart, kidneys, large intestine, liver, lungs, nasal cavity, pancreas, and small intestine) (Figure 4I). A common clinical feature among COVID-19 patients is respiratory symptoms. Some patients are accompanied by extrapulmonary symptoms such as cardiac injury, kidney injury, liver injury, ocular symptoms, and gastrointestinal symptoms (Lindner et al., 2020; Wichmann et al., 2020; Sridhar and Nicholls, 2021; Benvari et al., 2022; Brauninger et al., 2022; Chaurasia et al., 2022; Ramasamy, 2022). Among these, acute cardiac injury is a common extrapulmonary manifestation observed in COVID-19 patients (Chung et al., 2021; Liu F. et al., 2021). Figure 4I shows that the susceptibility to SARS-CoV-2 infection varies widely among different tissues, and the rate of disease progression also shows tissue-to-tissue heterogeneity. Lung, heart, bronchi, and nasal cavity show high susceptibility to SARS-CoV-2, which is consistent with a previous study highlighting the dominant pathological features of pulmonary and cardiovascular involvement (Falasca et al., 2020). On the other hand, the pancreas appears to be less susceptible to SARS-CoV-2 infection. Understanding tissue-specific mechanisms of COVID-19 infection and individual differences in disease progression will help identify novel targets for preventing disease progression in future studies.

## Discussion

COVIDanno is the first and unique database that systematically analyzed 745 SARS-CoV-2-infected and control (paired) samples from *in vitro* models and provides comprehensive annotations of downstream functional mechanisms. COVIDanno enables users to retrieve large-scale functional information and promotes understanding of virus-host interactions. In addition, COVIDanno provides a novel tool to help users predict the infection status for a given SARS-CoV-2-infected sample. In this study, we applied COVIDanno to explore anosmia symptoms, arrhythmia symptoms, and biomarkers in COVID patients, as well as to explore the susceptibility of 11 tissue types to SARS-CoV-2 infection. By applying COVIDanno, we identified multiple important genes associated with COVID-19 symptoms, such as UGT2A1, FGF12. Furthermore, we observed differences in immune responses between the upper respiratory tract and lungs during the early stages of SARS-CoV-2 infection. These findings are in line with previous reports. Comparing *in vitro* models to *in vivo* conditions in COVID patients can provide novel and effective insights to improve understanding of the relationship between host immune responses and disease progression. In order to keep COVIDanno at the forefront of the COVID-19 database, we will be constantly collecting and updating new data into our database. We believe that COVIDanno will be a valuable tool and platform for SARS-CoV-2-related research, facilitating a better understanding of pathogenesis, disease progression, biology, and improvement of therapeutic strategies.

## Data availability statement

The datasets presented in this study can be found in online repositories. The names of the repository/repositories and accession number(s) can be found in the article/Supplementary material.

## Author contributions

YF: software, data curation, conceptualization, and writing – original draft. MY: software and methodology. ZF: software and visualization. WZ: writing – review and editing. PK: conceptualization, project administration, and writing – review and editing. XZ: conceptualization, project administration, and writing – review and editing. All authors contributed to the article and approved the submitted version.

## Funding

YF and MY were supported by the 1·3·5 projects for disciplines of excellence–Clinical Research Incubation (2019HXFH022), Center of Excellence-International Collaboration Initiative Grant (139170052), West China Hospital, Sichuan University and Sichuan Science and Technology Program (2022YFS0228). ZF, WZ, and XZ were supported by NIH R01GM123037, U01AR069395-01A1, R01CA241930, and NSF 2217515. PK was supported by NIH R35GM138184.

## Conflict of interest

The authors declare that the research was conducted in the absence of any commercial or financial relationships that could be construed as a potential conflict of interest.

## Publisher’s note

All claims expressed in this article are solely those of the authors and do not necessarily represent those of their affiliated organizations, or those of the publisher, the editors and the reviewers. Any product that may be evaluated in this article, or claim that may be made by its manufacturer, is not guaranteed or endorsed by the publisher.
