# Supplementary material for: COVIDanno, COVID-19 annotation in human
Source: Front Microbiol. 2023 Jul 11;14:1129103. doi: 10.3389/fmicb.2023.1129103 (PMC10366449; doi:10.3389/fmicb.2023.1129103)
Supplement: Supplementary file 3 [file Data_Sheet_1.docx]

**COVIDanno, COVID-19 annotation in human**

Yuzhou Feng ^1, 2^, Mengyuan Yang ^3^, Zhiwei Fan ^4, 5^, Weiling Zhao ^4^, Pora Kim ^4, *^, Xiaobo Zhou ^4, 6, 7, *^

^1^ West China Biomedical Big Data Center, West China Hospital, Sichuan University, Chengdu 610041, China.

^2^ Med-X Center for Informatics, Sichuan University, Chengdu 610041, China.

^3^ School of Life Sciences, Zhengzhou University, Zhengzhou 450001, China.

^4^ Center for Computational Systems Medicine, School of Biomedical Informatics, The University of Texas Health Science Center at Houston, Houston, TX 77030, USA.

^5^ West China School of Public Health and West China Fourth Hospital, Sichuan University, Chengdu 610041, China.

^6^ McGovern Medical School, The University of Texas Health Science Center at Houston, Houston, TX 77030, USA.

^7^ School of Dentistry, The University of Texas Health Science Center at Houston, Houston, TX 77030, USA.

*Corresponding author(s).

E-mail:[Xiaobo.Zhou@uth.tmc.edu](mailto:Xiaobo.Zhou@uth.tmc.edu)(Zhou X), [Pora.Kim@uth.tmc.edu](mailto:Pora.Kim@uth.tmc.edu)(Kim P).

[**Supplementary figures**](https://oup.silverchair-cdn.com/oup/backfile/Content_public/Journal/bib/19/2/10.1093_bib_bbw109/2/bbw109_supplementary_data.docx?Expires=1596136237&Signature=KdVDacEa6cXQJcrLkgzRA1L8c1RDE3GDTkSTHbjgN9aKY5exIGcV7Kmpbdy8qbZJq7WsGdLZWBh0T6ITgm3feiY3xSYXqmqzXP9Vn5Z39xSX5UiCgUs8SWklHeGXrzaZ-iQPnfzJN0N~VjOEc~TmQHCj8yTvGZgBfmdnlSu97oUuXGMKV5Wi2VuNLiA4y3Yrxg9j9iC-nzTWMGWE7pZFJKHcH1dkPPAqimahHVcx4lePIHAMX5KCBSC0mlUH5ZIUl4ClWYEAJURQHX8MiOvAvoFg3gkOot9C373hcLEaBzwiN3YPCSEcI1PQM0YR~NecAo2tqdBu8Olz0gmxxP9WrA__&Key-Pair-Id=APKAIE5G5CRDK6RD3PGA)


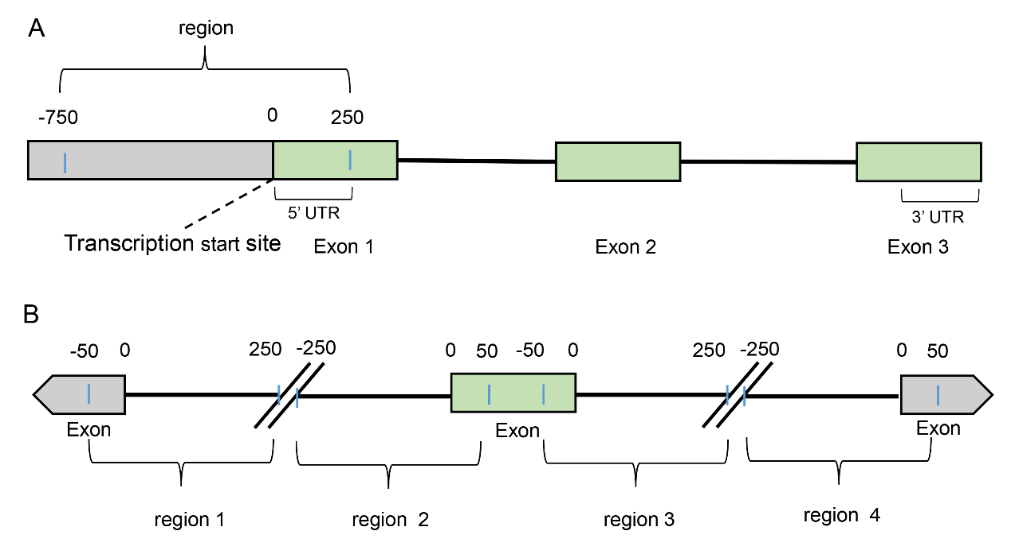


**Figure S1. The binding sites of genetic regulatory networks.** (A) Transcription factor binding site. (B) Binding sites for 4 RNA-binding proteins.


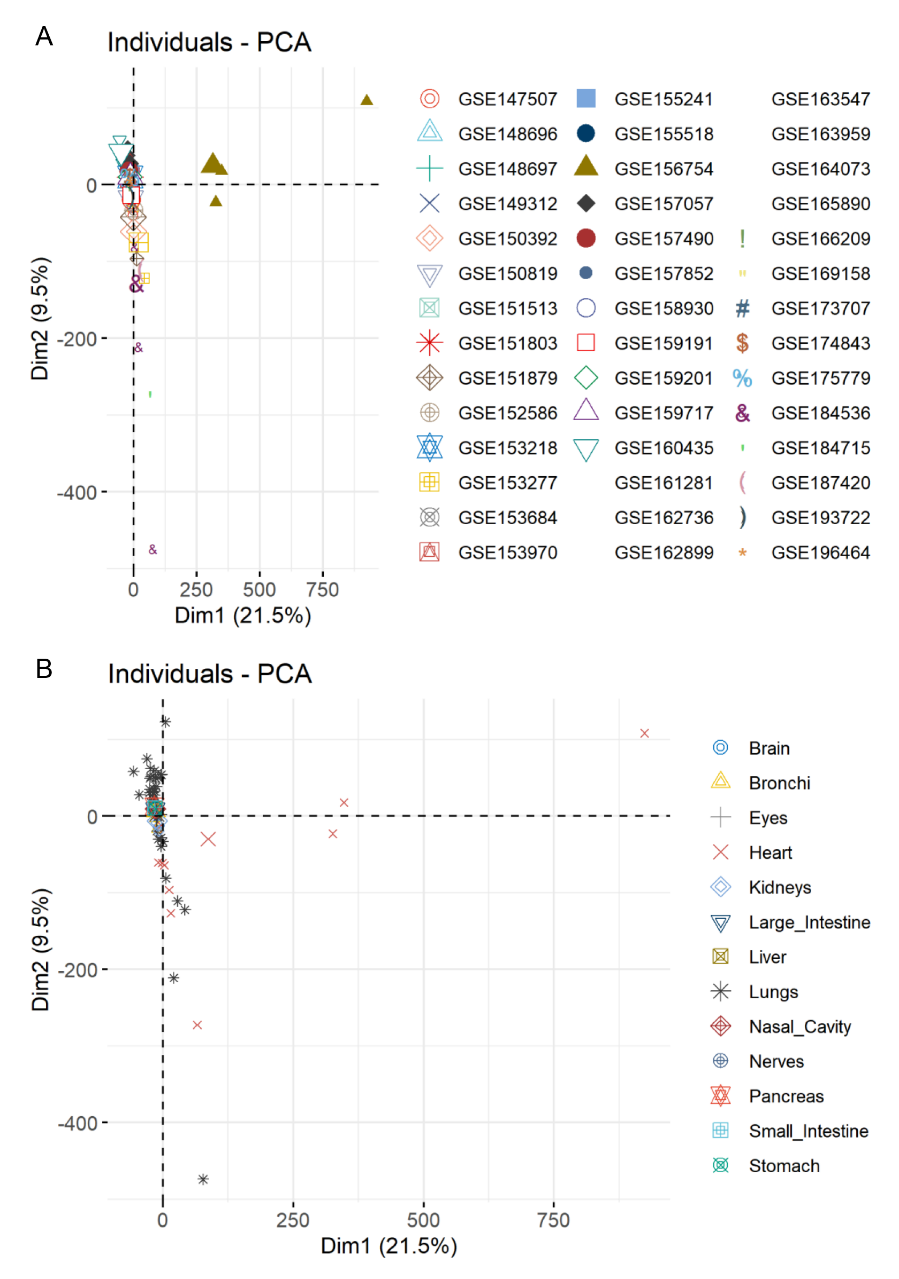


**Figure S2. The PCA plot** (A) Principal component analysis to investigate batch effects of 132 datasets using DEGs. (B) Principal component analysis to investigate tissue difference of 132 datasets using DEGs.


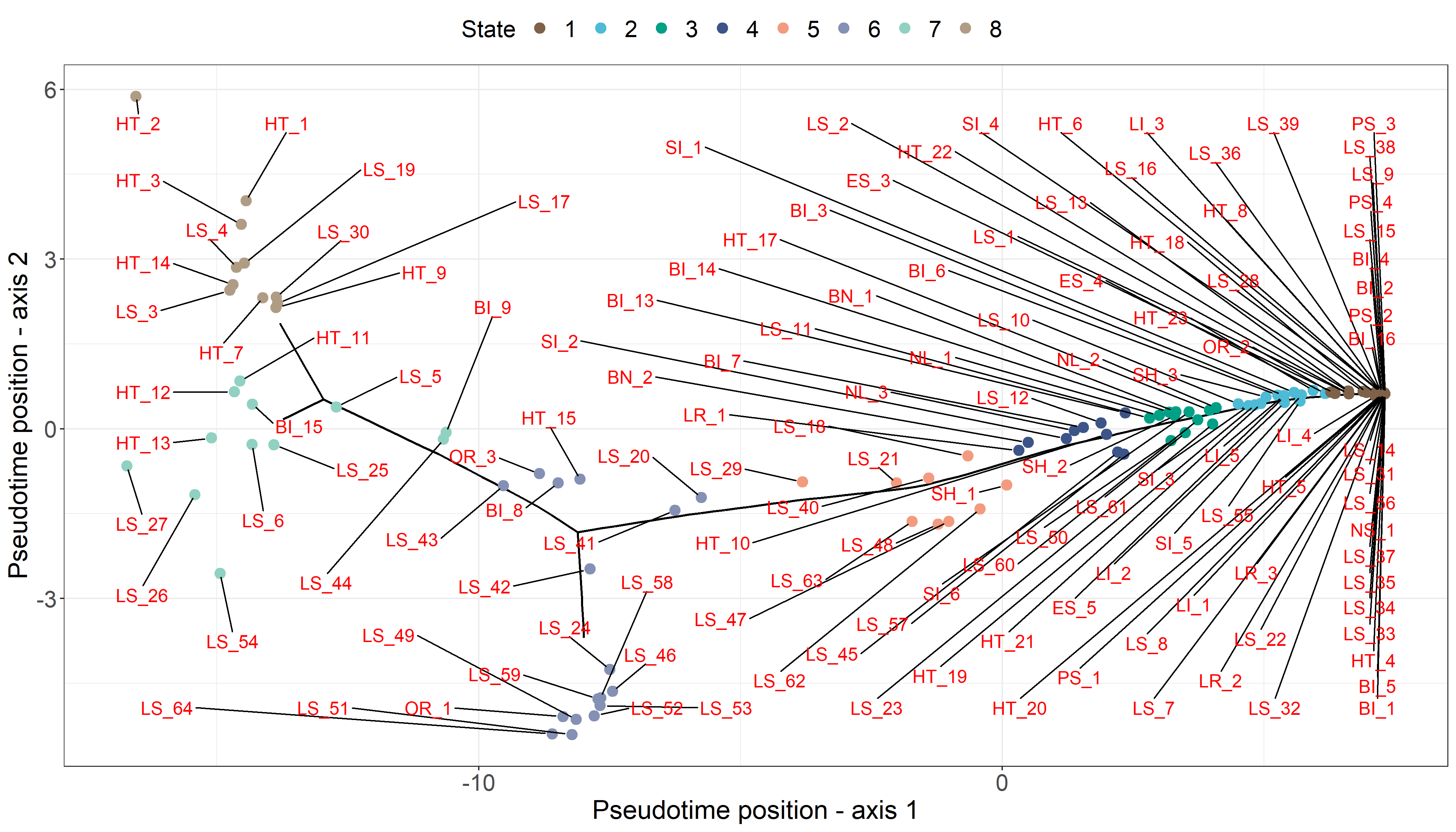


**Figure S3. Overview 132 datasets used in tool development.**


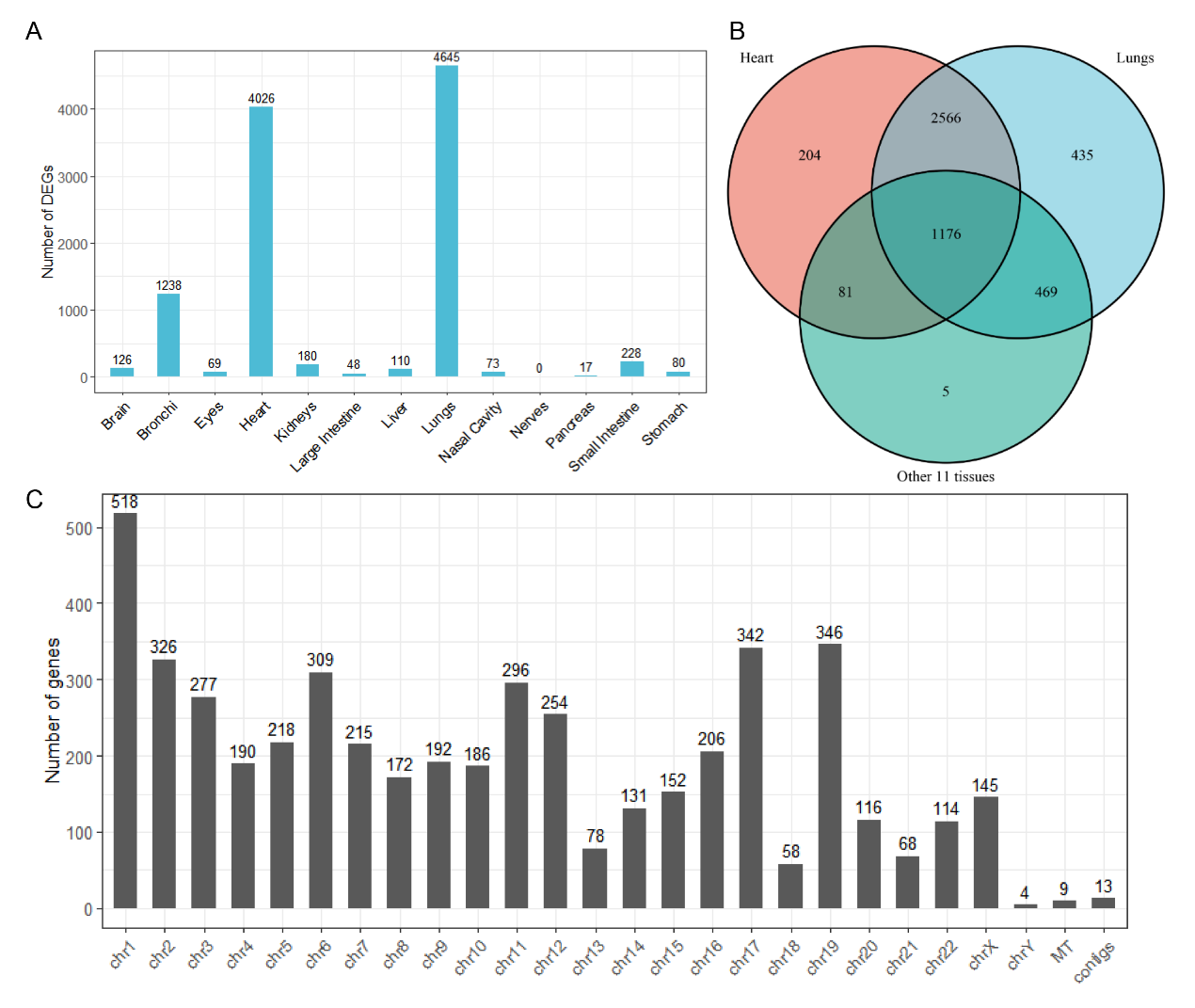


**Figure S4. The distribution of 4,935 DEGs.** (A) The distribution of 4,935 DEGs across 13 tissue types (B) The Venn diagram of 4,935 DEGs showing the overlaps across tissues. (C) The genomic distribution of 4,935 DEGs.


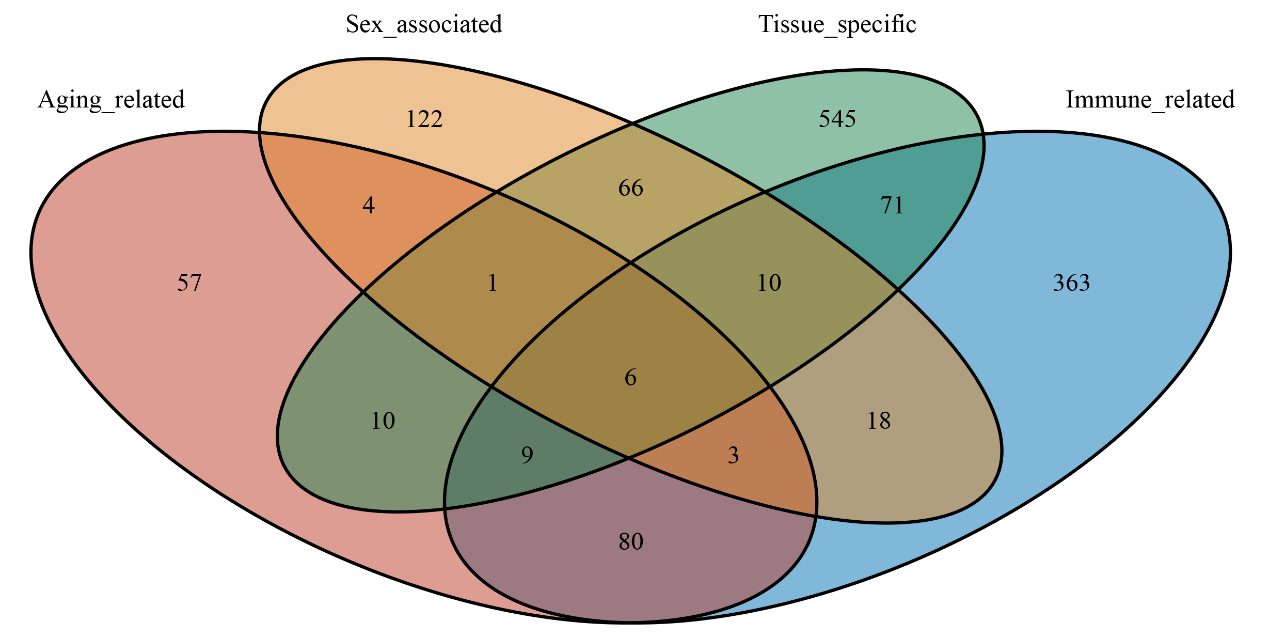


**Figure S5. Venn plot of annotated gene groups (immune relatedness, sex relatedness, aging relatedness, and tissue specificity)**


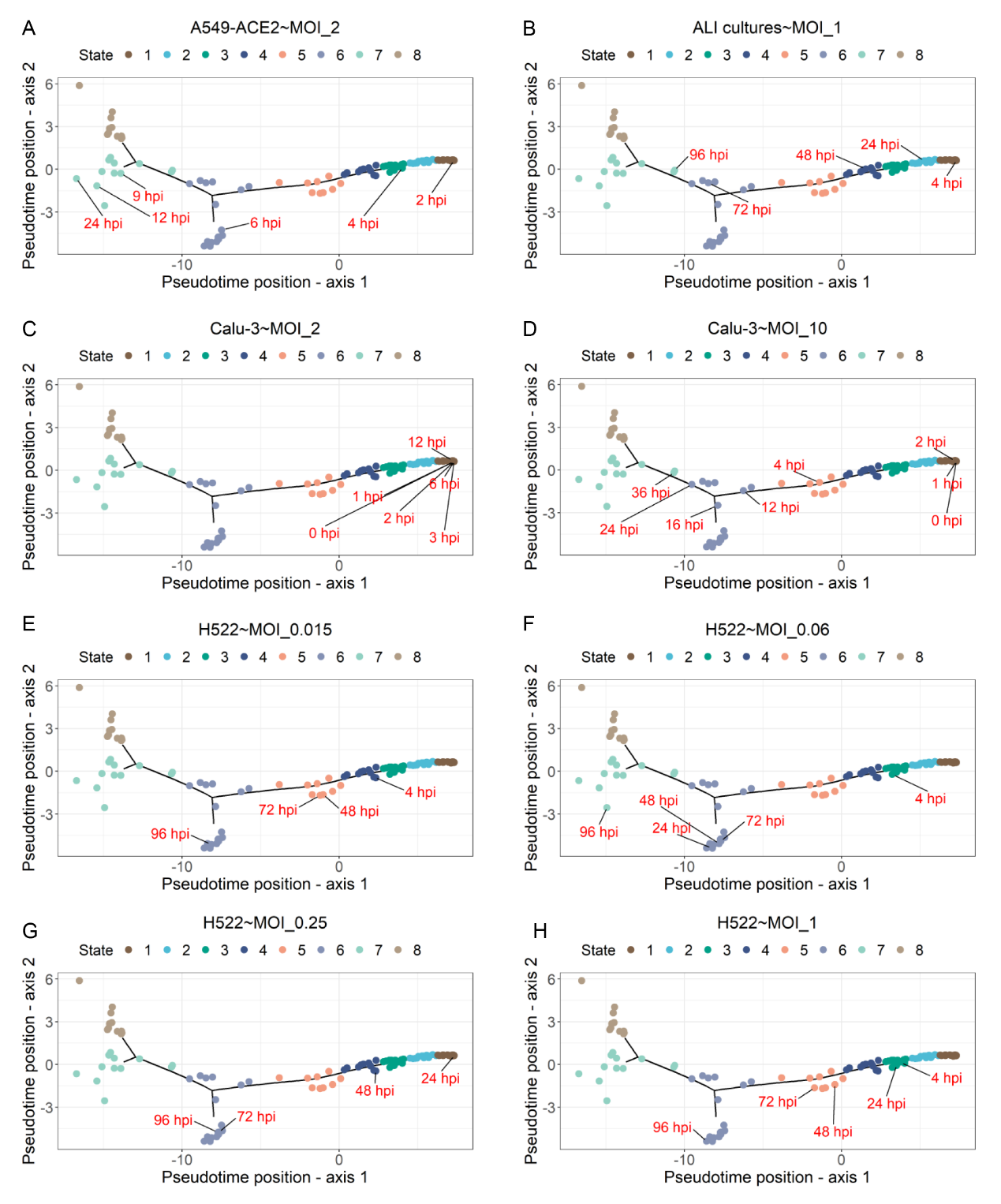


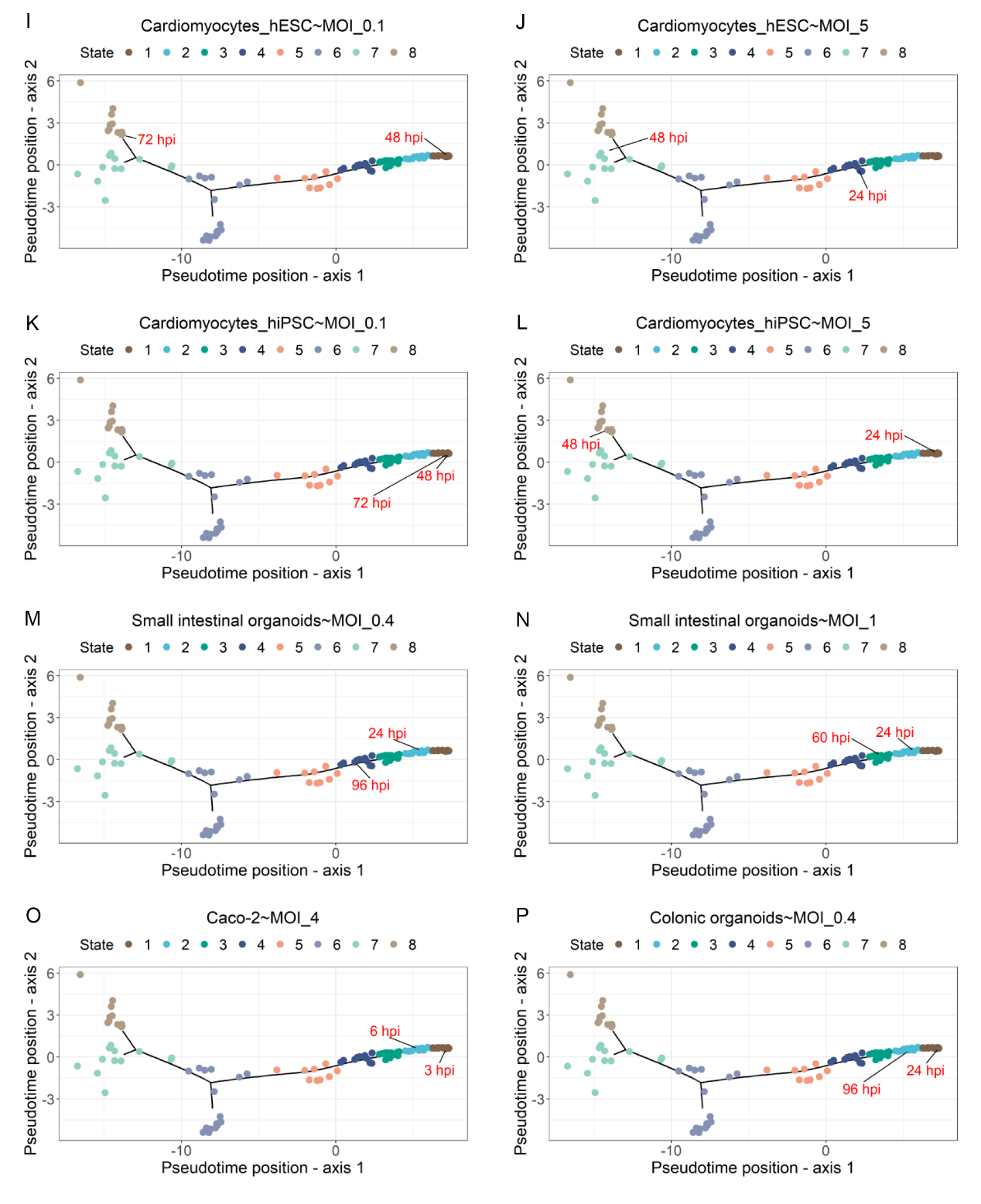


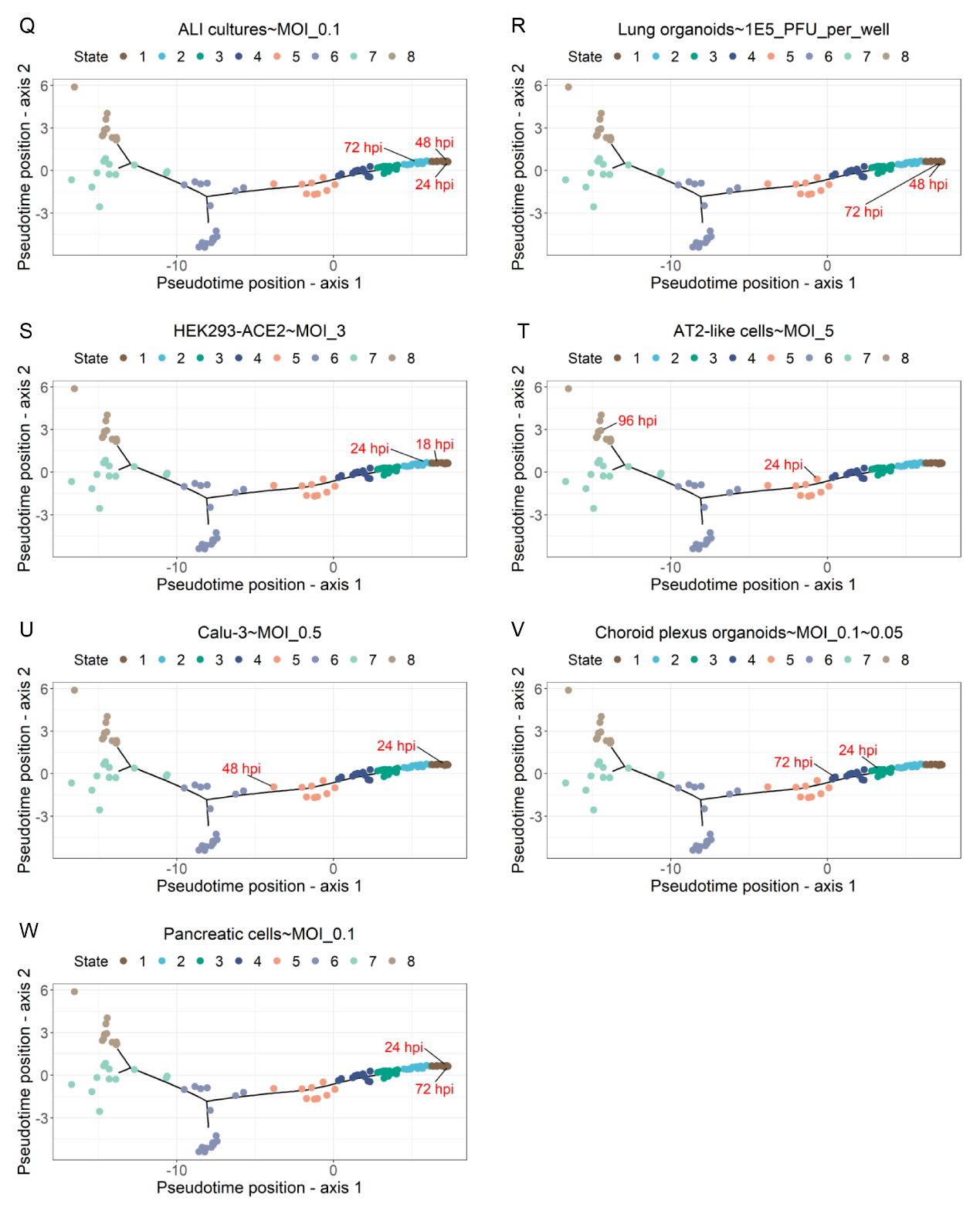


**Figure S6. The changes of infection severity over real infection time in experiments in the same sub-tissue type and multiplicity of infection value.** GEO accession number: (A) GSE184536. (B) GSE158930. (C) GSE151513. (D) GSE157490. (E) GSE163547. (F) GSE163547. (G) GSE163547. (H) GSE163547. (I) GSE162736. (J) GSE162736. (K) GSE162736. (L) GSE162736. (M) GSE159201. (N) GSE149312. (O) GSE162899. (P) GSE159201. (Q) GSE175779. (R) GSE157057. (S) GSE159191. (T) GSE153277. (U) GSE166209. (V) GSE157852. (W) GSE165890.


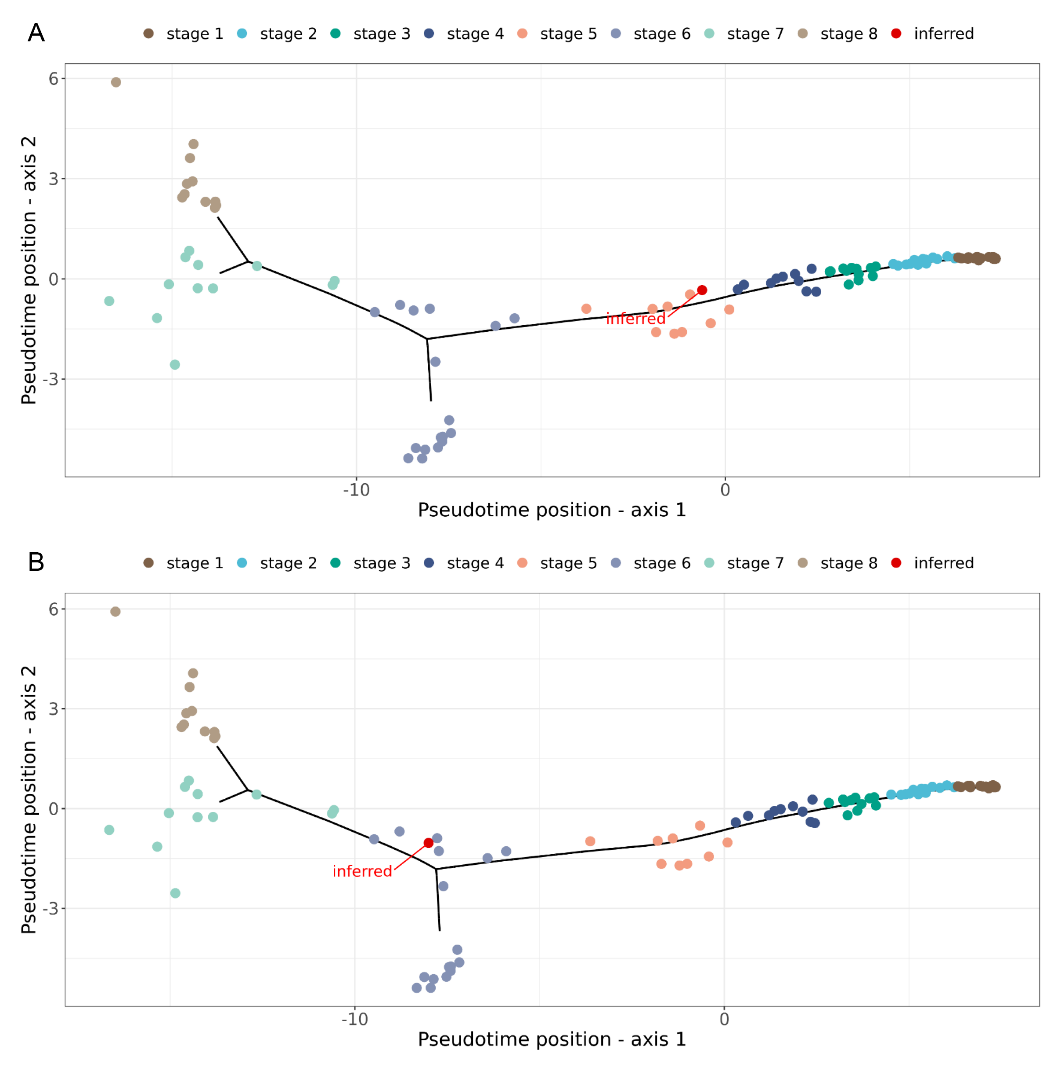


**Figure S7. Applying tool to explore datasets BI_10 and BI_11 in GSE196464 generated from SARS-CoV-2-infected cells in vitro.** (A) BI_10, 24 hours post-infection. (B) BI_11, 72 hours post-infection.


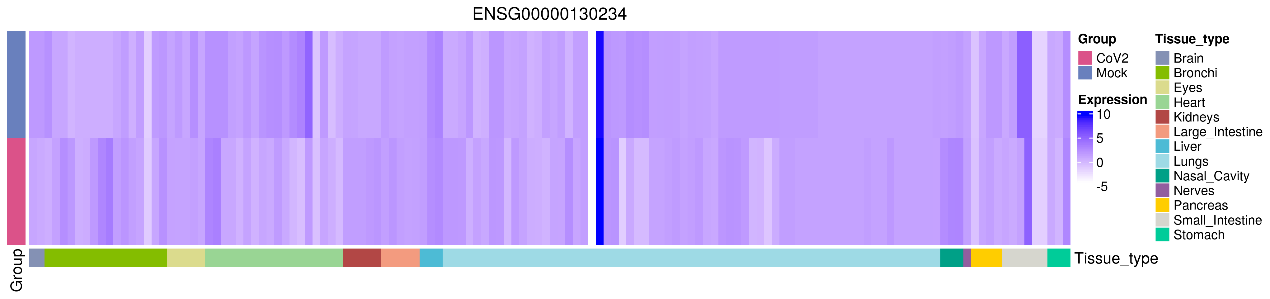


**Figure S8. Gene expression landscape of ACE2 across 136 datasets.**


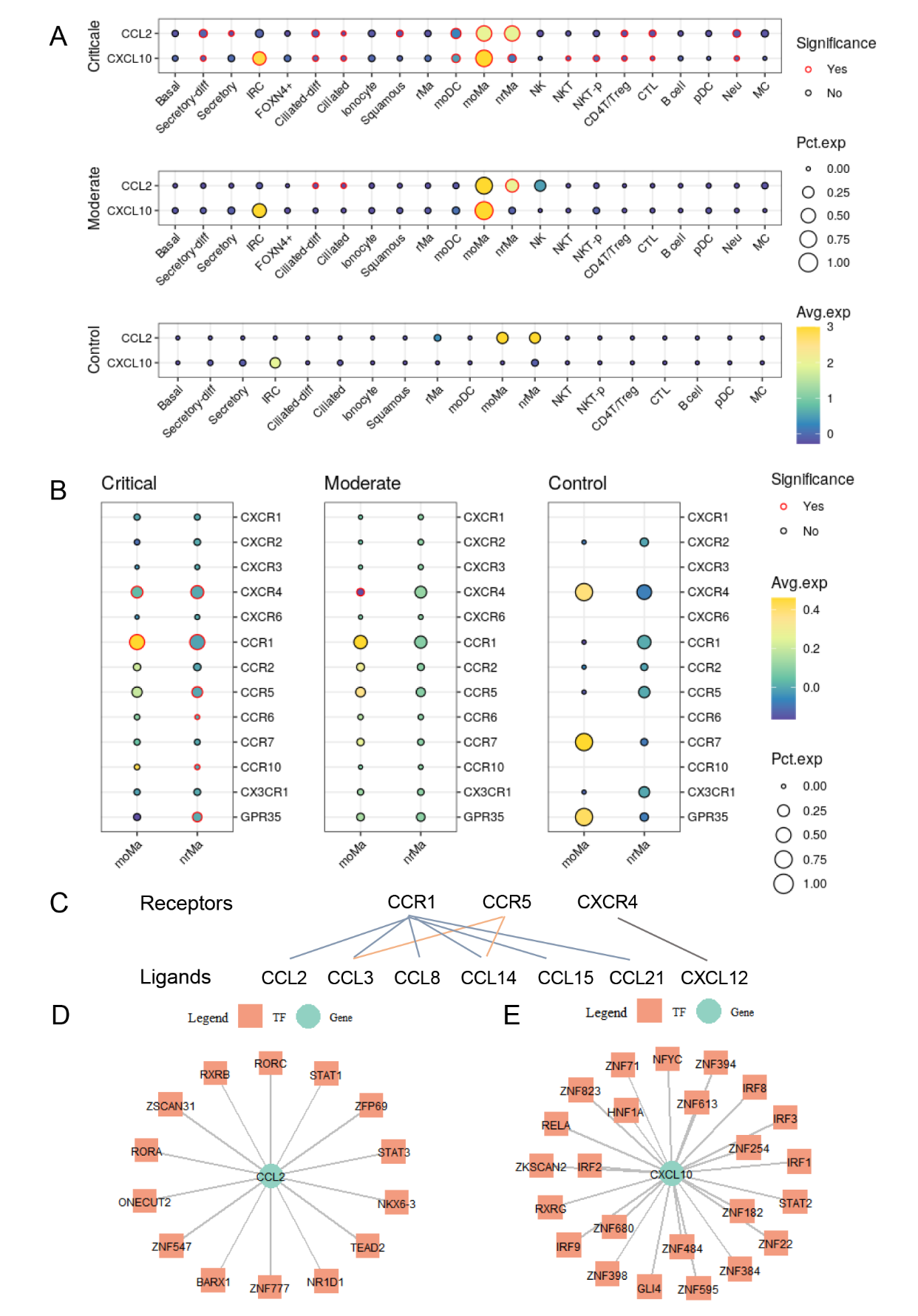


**Figure S9. Application of COVIDanno to explore the biomarkers associated with disease severity of COVID-19 in the respiratory tract.** (A) Expression of chemokines. (B) Expression of receptors. (C) Ligand–receptor pairs. (D) TF-gene regulatory network of CCL2 with top 30 TFs in lung tissues (A549, Calu-3, H522, Lung_organoids, Lung_tissues). (E) TF-gene regulatory network of CXCL10 with top 30 TFs in lung tissues (A549, Calu-3, H522, Lung_organoids, Lung_tissues).


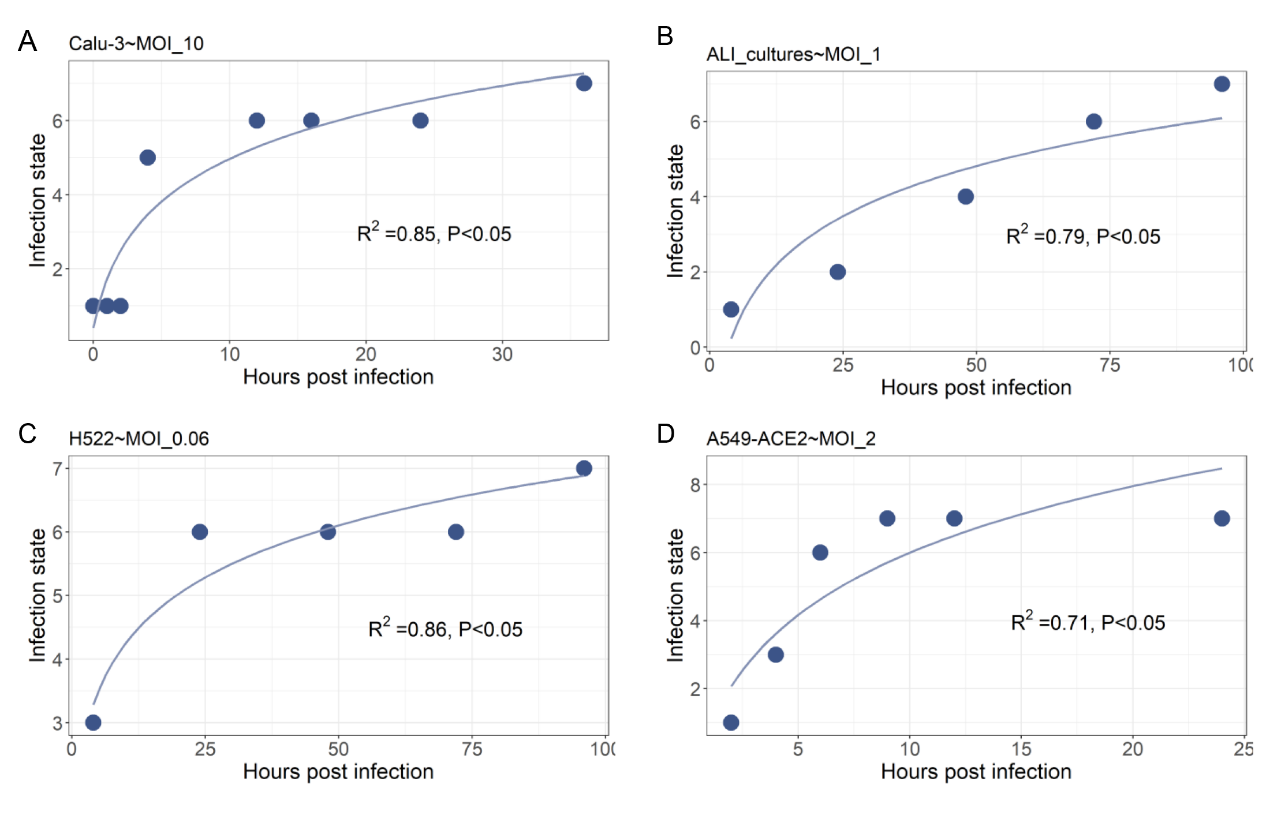


**Figure S10. The linear regression models with logarithmic transformations.** (A-D) using (Y = β0 + β1*log(X+1)) to fit prediction stage and post-infection time in experiments, (A) Calu-3 with MOI 10 from GSE157490 (B) H522 with MOI 0.06 from GSE163547 (C) ALI with MOI 1 from GSE158930 (D) A549-ACE2 with MOI 2 from GSE184536
